# A retrospective epidemiological cohort study of ankle fractures in children and teenagers

**DOI:** 10.1177/18632521231182424

**Published:** 2023-06-27

**Authors:** Christina Steiger, Giacomo De Marco, Céline Cuérel, Anne Tabard-Fougère, Moez Chargui, Romain Dayer, Dimitri Ceroni

**Affiliations:** Pediatric Orthopedics Unit, Pediatric Surgery Service, Department of Women, Child, and Adolescent, University Hospitals of Geneva, Geneva, Switzerland

**Keywords:** Pediatric, ankle fracture, epidemiologic, sports injuries, low-energy trauma

## Abstract

**Background:**

Ankle fracture is one of the most frequent pediatric lower-limb fractures and may result in serious complications.

**Objective:**

This study aimed to determine the epidemiology of ankle fractures, defining fracture types, treatments, and complications in a pediatric population below 16 years old.

**Methods:**

Medical records of all the ankle fracture patients treated in our hospital during 2004–2020 were retrospectively reviewed. Data regarding age, sex, mechanism of injury, fracture type, treatment modalities, and complications were collected.

**Results:**

We examined records involving 328 children with 331 ankle fractures, with a ratio of 1:2 male per female. Mean annual prevalence was 24.3 per 100,000 children. Mean patient age was 11.2 ± 4.2 years, with 75.3% of them aged over 10 years. Sports activities accounted for the largest percentage of fractures (162 cases; 49.4%), followed by falls (67; 20.4%) and road traffic accidents (37; 11.3%). Physeal fractures were the most frequent type of lesion (223 cases). Most ankle fractures (60%) were managed using closed reduction and casting; for the remaining 40% of cases, fracture fixation was performed after closed or open reduction to correct the articular step-off and ensure the anatomical restoration of the physis. The main ankle fracture complication was premature growth arrest (12.1% of all physeal fractures).

**Conclusion:**

Pediatric ankle fractures primarily affect children older than 10 years. Most of these fractures were caused by sports injuries or low-energy trauma. The majority of these fractures are physeal, and the distal tibial physis is affected 10 times more frequently than the distal fibular physis.

**Level of evidence:**

Level III

## Introduction

Pediatric ankle fractures are defined as tibia and fibula fractures distal to the metaphysis in patients with open or closed physes. Ankle fractures are among the commonest lower-limb fractures in older children and teenagers, occurring more often in boys than girls, and represent around 5% of all fractures and 15%–20% of all physeal injuries in children, also making them the commonest pediatric lower-limb physeal injury.^1–4^ The majority of these fractures are caused by sports injuries or low-energy trauma, such as in basketball, soccer, or inline skating.^5–7^ Ankle fractures account for up to 40% of all fractures in skeletally immature athletes.^8^ Fractures of the distal tibial physis have among the highest complication rates of all physeal injuries, with the risk of premature physeal arrest, bar formation, angular deformity, and articular incongruity.^9^ Indeed, growth arrest rates of up to 66.7% have been reported in the literature on injured distal tibial physes.^10–12^ By contrast, distal fibular physeal fractures are associated with a relatively low risk of long-term complications.^13^ Since the child’s ankle with incomplete ossification presents distinct mechanical and biological properties compared to skeletally mature patients, children will require specific treatment to preserve and monitor their physes. Although many reports have been published about the various options for treating pediatric ankle fractures, very few studies have focused on their demographic and etiological characteristics. Thus, the present study aimed to assess the ankle fractures of children and teenagers below 16 years in a single Swiss health district, paying particular attention to epidemiology, fracture patterns, treatments, and complications.

## Materials and methods

After ethics approval from the Review Board, we retrospectively reviewed the medical charts of all patients younger than 16 years admitted to our institution for ankle fractures between January 2004 and December 2020. Only patients with a follow-up of more than 2 years were included in this study. The Children’s Hospital is a 111-bed tertiary pediatric hospital providing specialized medical services for pediatric trauma to the region’s 80,000 children from 0 to 15 years old.

Data were collected on patient demographics, month of admission, mechanism of injury, and circumstances of trauma. Fractures were classified considering their location (epiphyseal, physeal, metaphyseal), their Salter–Harris classification for physeal fractures, and, if open, their Gustilo–Anderson classification. The Dias–Tachdjian classification of ankle fracture injury mechanisms was used for children with an open physis. The mechanisms of injury among teenagers were evaluated considering the Lauge–Hansen classification of ankle fractures, taking into consideration both the foot position at the time of injury and the direction of the forces applied. A pre-treatment computed tomography (CT) scan was performed in case of displaced intra-articular ankle fractures for investigating the interfragmentary gap and for defining epiphyseal fragments.

### Data analysis and statistical analysis

Data were categorized using patients’ demographic characteristics, epidemiological characteristics, fracture-specific information including fracture locations and types, and complication types. Findings were also analyzed considering the season in which the trauma occurred. In the northern hemisphere, spring begins on 1 March, summer on 1 June, autumn on 1 September, and winter on 1 December. All the data were tabulated using Excel spreadsheets, and statistical analyses were performed using SAS statistical software.

## Results

A total of 328 children with 331 ankle fractures were included in the study, with 178 fractures in male patients (54.3%), giving a ratio of 1:2 male fractures per female fracture. Three cases involved both ankles being fractured. The mean patient age was 11.2 ± 4.2 years; 81 children (24.7%) were younger than 10 years old, and the remaining 247 (75.3%) were older (10–15 years).

### Circumstances of injury

The mechanism of injury was unknown in three cases; the remaining 325 cases are shown in Table 1. Fractures due to sports activities made up 162 cases (49.4%), followed by 67 falls (20.4%) and 37 road traffic accidents (11.3%). Soccer was responsible for 25.3% of all fractures (41 cases); the other sports responsible for fractures are listed in Table 2. Of the 37 road traffic accidents, 26 patients were injured as pedestrians (70.3%) and 28 had been hit by a car (75.7%). In 11 cases, the patients had been riding another vehicle (5 bicycles, 5 motorcycles, and 1 quadbike) when the trauma occurred.

**Table 1.** Ankle fracture mechanisms of injury (n = 328).

	Number of cases (%)
Sports activities	162 (49.4)
Falls	67 (20.4)
Road accidents	37 (11.3)
Slipping, torsion, jumps from a height	32 (9.8)
Outdoor play	15 (4.6)
Foot caught in a bicycle wheel	7 (2.1)
Crushing trauma	5 (1.5)
Unknown trauma	3 (0.9)

**Table 2.** Sports activities responsible for ankle fractures (n = 162).

	Number of cases (%)
Soccer	41 (25.3)
Skateboard	22 (13.6)
Scooter	17 (10.5)
Gymnastics	16 (9.9)
Horse-riding	13 (8.0)
Trampoline	12 (7.4)
Skiing	9 (5.6)
Ice skating	5 (3.1)
Basketball	5 (3.1)
Sledging	4 (2.5)
Roller skating	3 (1.9)
Snowboarding	3 (1.9)
Motocross	2 (1.2)
Rugby	2 (1.2)
Badminton	2 (1.2)
Windsurfing	1 (0.6)
Acrobatic rock “n” roll	1 (0.6)
Parkour	1 (0.6)
Wakeboard	1 (0.6)

### Seasonal variations

Autumn was the peak season for pediatric ankle fracture diagnoses (100 cases), followed by spring (84 cases) and winter (82 cases). Unexpectedly, the smallest number of cases occurred in summer (62 cases).

#### Fracture types

Of the 331 ankle fractures, 163 cases involved lesions of the tibia alone, 44 cases involved lesions of the fibula alone, and 124 cases involved lesions to both the tibia and fibula. Thus, of the 455 lesions in total, there were 287 tibial and 168 fibular fractures. Physeal fractures were the commonest lesion (223 cases), more frequently to the distal tibia (203 cases) than to the distal fibula (20 cases). Physeal fractures were categorized as Salter–Harris type I (SH-I) lesions in 16 cases, SH-II in 121 cases, SH-III in 46 cases, and SH-IV in 40 cases (Table 3). Physeal fractures affected children 10 years old or more (195 cases) than children younger than 10 years old (28 cases). Metaphyseal and diaphyso-metaphyseal fractures were the second most frequently reported lesions (176 cases), more frequently found on the fibula (111 cases) than on the tibia (65 cases). Finally, the remaining 56 lesions were malleolus avulsion fractures. Any displacement of articular fragments was assessed using conventional X-rays or CT scans. In 107 patients, displacement of articular fragments was assessed using CT scans and reached an average of 3.1 mm. Articular step-off ranged between 0.5 and 21 mm; it was inferior to 2 mm in 32 cases; in the remaining cases, the articular gap was equal or superior to 2 mm.

**Table 3.** Types of ankle fracture (n = 331).

	Number of cases
Lesion of the tibia only	163
Lesion of the fibula only	44
Lesions to both the tibia and fibula	124
Physeal fractures	223
Distal tibia	203
Distal tibula	20
Classification physeal fractures	
Salter–Harris type I	16
Salter–Harris type II	121
Salter–Harris type III	46
Salter–Harris type IV	40
Metaphyseal and diaphyso-metaphyseal	176
Fibular diaphyso-metaphyseal	111
Tibial diaphyso-metaphyseal	65

#### Mechanisms of injury

By assessing our patient cohort’s ankle fracture radiographs, we estimated that the foot was in supination at the time of injury in 258 cases and in pronation in 69 cases. Direction of the forces exerted on the foot is listed in Table 4.

**Table 4.** Ankle fracture mechanisms of injury (n = 331).

	Number of cases
Position of the foot at the time of injury	
Supination	258
Pronation	69
Supination and external rotation	80
Supination and plantar flexion	79
Supination and inversion	78
Supination and adduction	21
Pronation and external rotation/eversion	69
Dorsal flexion with axial compression	6

#### Treatments and complications

Two hundred fractures were treated using closed reduction and casting. Of the 131 lesions requiring surgical treatment, 88 fractures were treated by closed reduction combined with either percutaneous wiring or cannulated screw fixation or external fixation. A further 43 cases required an open reduction combined with either cannulated screw fixation or plate and screw fixation. Nevertheless, 47 complications were identified (Table 5), including 27 growth arrest that resulted in 12 cases of angular disturbance, 10 cases of segment length inequality, and 5 cases where residual growth potential was insufficient to express a length or axis disorder. Of the 27 cases with effective growth disturbance, 16 were consecutive to an orthopedic treatment (closed reduction and cast immobilization), whereas 11 were subsequent to a surgical treatment. The rate of premature physeal closure was 14.6% for SH-I and SH-II fractures and 9.3% for SH-II and SH-IV fractures. Of the 27 cases resulting in a growth disorder, 9 (33%) required a corrective surgery; 2 of these were intended to correct a length problem, while the remaining 7 were intended to resolve an axis disorder.

**Table 5.** List of complications (n = 47).

	Number of cases
Skin disorders	7
Peripheral neuropathy	4
Impingement due to osteosynthesis material	5
Loss of the fracture reduction	2
Tibio-peroneal synostosis	2
Ankle stiffness	1
Growth disturbances	27
Segment length inequality	10
Angular disturbance	12
Without clinical repercussions	5
Rate of growth arrest SH I–II fractures	14.6%
Rate of growth arrest SH III–IV fractures	9.3%

SH: Salter–Harris.

## Discussion

Despite the frequency of ankle fractures in pediatric populations, few published studies have focused on epidemiological data. In addition, these studies were usually based on data from pediatric hospital discharge databases or trauma registries. Since the present research was a retrospective, single-center study based on electronic medical records, we are very confident in the accuracy of our findings.

Findings demonstrated that the reported incidence of childhood ankle fractures (24.3/100,000/year) was almost half the 42/100,000/year reported in an earlier population-based cohort study.^14^ However, the lower incidence of pediatric ankle fractures reported in this study may be because many private clinics also provide care for children with ankle fractures in our region.

Contrary to the classic description of all types of fractures affecting pediatric populations, we did not detect a bimodal age distribution.^8^ Our findings seemed to confirm that pediatric ankle fractures are commonest between 8 and 15 years old, as the growth plates are beginning to fuse.^1,5–7,14,15^ Differences might also be explained by increasing weight, muscular force, and activity with age, factors which contribute to higher energy levels in trauma among older children.

We also detected only a slight male predominance in the incidence of pediatric ankle fractures, with 1:2 male fractures for each female fracture: previous studies showed twice as many ankle fractures in boys than in girls.^16–19^ Many explanations for the higher incidence of male ankle fractures have been postulated—especially for teenagers—mostly associated with greater participation in outdoor play, sports, and reinforced behaviors.^20^ Indeed, the majority of sports-related injuries in our study were in boys, showing that participation in sports tends to increase fracture risks for boys and decrease them for girls.^21^ Furthermore, males’ more hot-headed temperaments when they start to drive motorized vehicles leads presumably to an increased fracture risk in boys. The peak in road accidents among children older than 14 years old has also been associated with reaching driving age.^22^

Seasonal variations in ankle fractures were somewhat different from what one might have expected. Fractures are generally recognized as being more frequent during the summer, when children are out of school and doing outdoor physical activities.^23,24^ The most consistent climatic factor has appeared to be hours of sunshine, and the average number of fractures in the summer was estimated to be 2.5 times that in winter.^25^ Contrary to this, our study demonstrated that more ankle fractures occurred in autumn. The lower frequency of recorded summer fractures might be explained by the fact that significant numbers of children leave the city during the holidays, and many may sustain fractures outside our hospital catchment area, thus, not being registered in our hospital.

Supination was the commonest initial foot position at the time of injury, registered for 77.9% of ankle fractures. External rotation, plantar flexion, and inversion were the main directions of force applied on those supinated feet, in roughly equivalent proportions. When the foot’s initial position was pronation (22.1%), external rotation and eversion were the main directions of force applied to the foot.

The present study showed that the commonest mechanisms of injury were linked to sports accidents (49.4 %), followed by low-energy falls (20.4%) and road traffic accidents (11.3%). Soccer alone was responsible for a quarter of all ankle fracture injuries, and over a quarter were due to different roller sports accidents. These observations validated the conclusions of previous studies, which had shown that basketball, soccer, football, and scooters were the commonest activities associated with pediatric ankle fractures.^5–7^

Our study also revealed that 64.7% of pediatric ankle fractures were physeal (223 cases), with the distal tibial physis being affected 10 times as often as the distal fibular physis. The occurrence of physeal fracture was closely correlated with patient age as, in this series, 87.4% of physeal fractures affected children 10 years old or more. Under 10 years old, most fractures occurred in the metaphysis. Salter–Harris type II (SH-II) was the commonest type of physeal ankle fracture, accounting for 54.3% of them. SH-II is generally regarded as the commonest physeal ankle fracture, accounting for 32%–40% of fractures, followed by SH-III (25%), SH-IV (up to 25%), SH-I (3%–15%), and SH-V (less than 1%).^7,26^ This physeal fracture classification is crucial since it is believed to determine the prognosis, which appears to be more favorable for SH-I and SH-II than for SH-III, which is, in turn, better than for SH-IV.

Almost a third of the ankle fractures (105 cases) in this series necessitated a pre-treatment CT scan evaluation to investigate the fracture’s configuration, especially the gap, and this was both for the physis and potential epiphyseal fragments. Our investigation showed there to be a mean gap of 3.1 mm. This is crucial because higher rates of physeal arrest (60% versus 17%) could be seen at the physis if a residual gap >3 mm is present, even for SH-I and SH-II fracture patterns; thus, open reduction to remove entrapped periosteum is recommended in these settings.^10^ Clinicians dealing with physeal fractures should consider a CT scan evaluation, especially when there is any doubt remaining about the size of the gap before or after a closed reduction on a conventional X-ray examination.

Our results confirmed that many ankle fractures could be treated simply since 60% of this series were managed using closed reduction and casting. It is important to remember that the repeated or delayed manipulation of physeal fractures should be avoided so as to prevent additional damage to the physis and the subsequent risks of premature closure.^10,26^ The present study also confirmed that intra-articular fractures and physeal fractures should be reduced anatomically to restore joint surface congruency, correct angular limb deformity, and decrease the complications’ rate. Indeed, it is unanimously accepted that articular step-off should be less than 1–2 mm.^27^ In 39.4% of our cases, fixation was performed after either satisfactory manipulation or open reduction. Open reduction combined with internal fixation or percutaneous stabilization after a satisfactory closed reduction are the recommended methods for treating displaced intra-articular fractures. The goals for both methods are to establish an articular step-off of less than 1–2 mm^27^ while simultaneously ensuring an anatomical reduction of the physis to facilitate physeal growth. Our findings demonstrated that closed reduction should be successful for SH-I and SH-II fracture patterns, whereas displaced SH-III and SH-IV fracture patterns require anatomical reduction, stable internal fixation, restoration of the articular surface, and an anatomical reduction of the physis.

Finally, our study found a low complication rate (14.3%). Growth arrest was only noted in 27 cases, which corresponded to a risk of premature physeal closure of 12.1% of total physeal injuries. With regard to the Salter–Harris classification, the rates of physeal arrest were 14.6% for SH-I and SH-II fractures and 9.3% for SH-III and SH-IV fractures. These levels of risk were within those of previous studies that showed overall risks of premature physeal closure ranging from 2% to 67% for SH-I and SH-II fractures and from 8% to 50% for SH-III and SH-IV fractures.^9,10,26,28–30^ We only found one case of premature physeal closure of the distal fibula, confirming the low risk of isolated fibular growth arrest.^30^ Contrary to popular belief, we noted that there are no more growth disorders in children depending on the severity of the fracture when applying the Salter–Harris classification. Our study results showed that the premature physeal closure rates are relatively the same between those of previous studies that showed overall risks of premature physeal closure ranging from 2% to 67% for SH-I and SH-II fractures and from 8% to 50% for SH-II and SH-IV fractures.^9,10,26,28–30^ Interestingly, growth disturbance was not more frequent after surgical treatment (11 cases) than after orthopedic conservative treatment (16 cases). Therefore, our study results seemed to confirm the theory that surgical management of SH III–IV physeal tibial fractures reduce the risk of premature physeal closure, but that this is not proven for physeal tibial fractures SH I–II.^31^

The present study’s main limitation was its retrospective observational nature. Its core strengths were its large cohort of children and adolescents and, above all, its detailed and precise medical records.

## Conclusion

The present study examines pediatric ankle fractures and reviews their epidemiology, fracture patterns, mechanisms of injury, treatment methods, and potential complications. Most of the fractures are caused by sports injuries or low-energy trauma. Around 60% of pediatric ankle fractures are physeal, and there are 10 times as many fractures of the distal tibial physis as of the distal fibula. We strongly recommend using CT scans to evaluate pediatric ankle fractures before treatment to investigate their configuration and especially the gap, both in the physis itself and between epiphyseal fragments.

More than 60% of fractures are probably accessible to conservative treatment with cast immobilization with or without reduction in general anesthesia. Premature physeal closure is the most significant complication found in our series in ankle fractures. However, this complication remains a rarer complication than expected, amounting to 12.1% of all physeal fractures. In that regard, premature physeal closure occurs more frequently after type I and II fractures than after type SH I–III and IV lesions. In addition, growth disturbance seemed to be more frequent after orthopedic conservative treatment than after surgical management.

Finally, the therapeutic impact of this complication is few since only a third of patients with growth disturbance had to be revised surgically.
